# Effects of Ferulic Acid on Meat Quality, Carcass Traits, Muscle Fiber Types, and Muscle Development in Shaziling Pigs

**DOI:** 10.3390/foods15122111

**Published:** 2026-06-11

**Authors:** Xiwen Zhang, Shuning Jin, Chenghuan Hu, Qi Luo, Yulian Li, Jianhua He, Yurong Zhao

**Affiliations:** 1 College of Animal Science and Technology, Hunan Agricultural University, Changsha 410128, China; cenyangdi@126.com (X.Z.);; 2 Xiangtan Livestock Breeding Station, Xiangtan 411100, China

**Keywords:** pig, ferulic acid, carcass characteristics, meat quality, muscle development

## Abstract

The mechanisms by which ferulic acid (FA) improves meat quality and promotes muscle development in pigs remain unclear. This study evaluated the effects of dietary FA on meat quality and muscle development in Shaziling pigs. A total of 108 pigs (30 kg ± 0.71 kg) were selected and randomly assigned to three groups, with six replicates per group and six pigs per replicate. Pigs were fed a corn–soybean meal basal diet supplemented with 0 mg/kg, 250 mg/kg, or 500 mg/kg of FA for 120 days. Results showed that FA reduced fat percentage, increased lean meat percentage and loin-eye area, enhanced redness and pH, and elevated muscle crude protein content. It also increased muscle fiber diameter and improved antioxidant enzyme activities (*p* < 0.05). The FA increased the levels of capric acid and methionine in the muscle, while reducing the content of stearic acid (*p* < 0.05). Furthermore, analysis of muscle development-related genes showed that FA upregulated the expression of *PI3K*, *AKT1*, *mTOR* and downregulated the expression of *FOXO1* and *MSTN* in the muscle (*p* < 0.05). Analysis of muscle fiber type and glucose metabolism-related genes revealed that FA upregulated the expression of *PGC-1α*, *MYH IIa*, *MYH I*, *HK2*, and *PFK* and downregulated the expression of *PKM* and *MYH IIX* in the muscle (*p* < 0.05). In conclusion, FA improves muscle development and meat quality in Shaziling pigs, possibly through modulation of genes associated with PI3K/AKT/mTOR signaling, enhancement of antioxidant capacity, and regulation of muscle fiber type and glucose metabolism.

## 1. Introduction

The global demand for high-quality pork necessitates nutritional strategies that enhance both lean growth and meat quality while moving away from synthetic additives [1]. In this context, dietary phytochemicals have emerged as viable alternatives, with ferulic acid (FA) gaining considerable attention. FA, a ubiquitous phenolic compound in plant cell walls, is recognized for its potent antioxidant, antimicrobial, and cytoprotective properties [2,3]. Valenzuela-Grijalva et al. found that FA supplementation can significantly improve growth performance and carcass characteristics, including increasing loin muscle area and reducing fat deposition, effects comparable to those induced by synthetic β-adrenergic agonists [4]. However, the translation of these benefits to indigenous Chinese pig breeds which possess distinct genetic predispositions for fat deposition and muscle fiber composition remains poorly understood. The Shaziling pig (*Sus scrofa domesticus*), a valued indigenous Chinese pig breed, typically exhibits superior meat flavor but often suffers from lower lean percentage and inconsistent quality traits compared to commercial hybrids [5]. Therefore, elucidating the precise molecular mechanisms through which FA improves pork quality and promotes skeletal muscle development in such breeds is critical for optimizing production systems tailored to local genetic resources.

The capacity of FA to improve pork quality is mechanistically linked to its modulation of cellular signaling, oxidative status, and muscle fiber plasticity. Central to muscle hypertrophy is the PI3K/AKT/mTOR signaling pathway, a master regulator of protein synthesis and myogenesis [6]. Although direct evidence in porcine muscle is still emerging, studies in skeletal muscle cell models indicate that FA can activate the IRS-1/Akt signaling cascade, thereby promoting glucose uptake and anabolic metabolism [7]. Concomitantly, FA-mediated downregulation of FOXO1, a negative regulator of muscle mass, further reinforces an anabolic environment [8]. Beyond protein accretion, the improvement of meat color and pH stability is largely attributed to FA’s robust antioxidant capacity. As a potent free radical scavenger, FA preserves postmortem muscle integrity by mitigating oxidative damage to lipids and proteins, which is particularly relevant under modern production stressors [9]. Furthermore, FA induces a favorable shift in muscle fiber type composition. By upregulating PGC-1α and promoting the expression of oxidative myosin heavy chain isoforms at the expense of glycolytic fibers, the FA enhances mitochondrial biogenesis and oxidative metabolism [10]. This fiber type transition is crucial for indigenous breeds, as it correlates strongly with improved water-holding capacity and the stabilization of postmortem pH decline, ultimately elevating the overall technological and sensory quality of the pork.

Despite the promising data derived from Western commercial lines, a significant knowledge gap persists regarding the efficacy and mechanistic action of FA in Chinese indigenous breeds like the Shaziling pig, which exhibit unique metabolic and myofiber characteristics [11,12]. The present study was therefore designed to address this gap by evaluating the effects of dietary FA supplementation over a 120-day finishing period in Shaziling pigs. Our investigation specifically aimed to explore whether FA supplementation is associated with changes in the expression of key components of the PI3K/AKT/mTOR signaling axis, enhancement of endogenous antioxidant enzyme activity, and transcriptional reprogramming of muscle fiber types and glycolytic enzymes. These findings offer a scientific foundation for the application of FA as a natural, functional feed additive to enhance both production efficiency and meat excellence in Shaziling and potentially other local pig populations.

## 2. Materials and Methods

### 2.1. Experimental Design and Feeding Management

One hundred and eight Shaziling pigs with an average initial body weight of 30 kg ± 0.71 kg were randomly assigned to three groups, each with six replicates and six pigs per replicate (half barrows and half gilts). The pigs were fed a corn–soybean meal basal diet supplemented with 0 mg/kg, 250 mg/kg, or 500 mg/kg of the FA, respectively. The adaptation period lasted 7 days, and the experimental period lasted 120 days. The basal diet was formulated according to the nutrient requirements for fatty-type growing finishing pigs specified in the Chinese Swine Feeding Standard (GB/T 39235-2020) [13]. The diet composition and nutrient levels are presented in Table S1. During the adaptation period, routine immunization and feed transition were carried out. Throughout the experiment, the pigs were managed by designated personnel. They were fed twice daily, at 8:00 a.m. and 4:00 p.m., following the principle of feeding to satiety without leftovers, and had ad libitum access to water. Feed consumption was recorded. The pens were kept clean and sanitary, with attention to proper ventilation, and the corridors of the pig house were disinfected regularly with a disinfectant. The condition of the pigs was observed daily, and daily work records as well as health records of the herd were maintained. The FA purity was >95% (Product Number: 537984) and it was purchased from Xi’an Tianguangyuan Biotechnology Co., Ltd., Xi’an, China.

### 2.2. Sample Collection

At the conclusion of the feeding trial, one barrow with the body weight closest to the pen mean was selected from each replicate to minimize within-pen variation. This animal was used as the experimental unit for subsequent analyses (*n* = 6 per group). The pigs were rested for 12 h prior to slaughter and stunned using electrical stunning (both temples and the precordial region, 90 V, 1.5 A, 2 s). Slaughter was performed in a licensed commercial abattoir (Xiangtu Slaughterhouse, Xiangtan, China) following standard animal welfare and meat inspection protocols. Cervical venous blood was drawn into blood collection tubes and allowed to stand for 30 min, then centrifuged at 3000 r/min for 15 min to obtain serum. The serum samples were stored at −80 °C until analysis. After exsanguination, carcasses were immediately eviscerated and split. No chilling was applied before the 45 min measurements; thereafter, carcasses were chilled at 4 °C for 24 h. The longissimus thoracis (LT) muscle was collected from the left side of the carcass between the 10th and 12th ribs. A portion of fresh LT muscle samples was immediately used for meat color and pH measurement at 45 min, and then stored at 4 °C for the same measurements at 24 h. Another portion was stored at 4 °C for the determination of moisture, crude protein, crude fat, shear force, drip loss, water loss ratio and cooking yield, amino acids, fatty acids, and physiological and biochemical parameters. Some muscle samples were stored at −80 °C until used for gene expression analysis. Meanwhile, a separate portion of LT muscle was cut into 0.5 cm × 0.5 cm × 0.5 cm cubes along the muscle fiber direction, fixed in 4% paraformaldehyde for 48 h, and then subjected to hematoxylin and eosin (H&E) staining for histomorphological analysis.

### 2.3. Carcass Traits

The body composition was analyzed through slaughter and dissection, following the method described by Xie et al. [14]. Briefly, following slaughter, the carcass was split; the head, feet, tail, and viscera (except the kidneys) were removed, and the carcass was weighed. The left side of the carcass was then separated into skin, bone, lean meat, and fat using a scalpel, and each component was weighed separately. Carcass yield (%) = (hot carcass weight/live body weight) × 100. Lean meat percentage (%) = (lean meat weight/total weight of skin, bone, lean meat, and fat) × 100. Fat percentage (%) = (fat weight/total weight of skin, bone, lean meat, and fat) × 100. Backfat thickness was measured using a digital caliper at the first, tenth, and last rib positions, and the average of these three measurements was used as the backfat thickness for each carcass [15]. The LT muscle area was determined at the left thoracolumbar junction. The maximum height and width of the LT muscle were measured using a digital caliper. Muscle height was measured from the dorsal to the ventral side, and width was measured perpendicular to the height. The cross-sectional area of the LT muscle was calculated using the formula: Loin-eye area (cm^2^) = Loin-eye area height × Loin-eye area width × 0.7.

### 2.4. Meat Quality and Muscle Chemical Composition

The left LT muscle was collected from each pig and allowed to bloom for approximately 20 min. The meat color at 45 min and 24 h postmortem lightness (L*), redness (a*), and yellowness (b*) were measured using a portable colorimeter (OPTO-STAR, MATTHAUS, Pöttmes, Bavaria, Germany) after preheating and calibration. The operator should have normal color vision. The measurement illuminant of the portable colorimeter is D65, and its aperture is 20 mm. The observer is the CIE 10° standard observer. The pH value was determined at 45 min and 24 h postmortem using a portable muscle pH meter (PH-STAR, MATTHAUS, Pöttmes, Bavaria, Germany) [16]. Shear force, drip loss, press loss, and cooking yield was measured according to the methods of An et al. [17]. Homogenized muscle samples were analyzed according to AOAC methods (2000) for crude fat (ether extract method 920.39), moisture (oven drying method 934.01) and protein content (Kjeldahl system with a 6.25 × N conversion factor to crude protein, method 954.01) [18]. After fixation for 48 h, LT samples were sequentially dehydrated, cleared, and embedded in paraffin. Sections were cut, deparaffinized, stained with H&E, and mounted. The staining kit was provided by Nanjing Jiancheng Bioengineering Institute, China. Following microscopic observation, muscle fiber cross-sectional area, number, and density were measured using Image J software (1.54r, https://imagej.net).

### 2.5. Medium- and Long-Chain Fatty Acids

Qualitative and quantitative analysis was performed using gas chromatography (8890, Agilent, Santa Clara, CA, USA). For each sample, 5 g of LT muscle sample was accurately weighed, dried for 48 h using a vacuum freeze dryer (GLZ-10, Shanghai, China), and then weighed again to determine the moisture content. The moisture content was then used to convert the fatty acid content to a wet muscle weight basis. Grind the freeze-dried pork sample, weigh approximately 0.5 g of the dry powder, and place it in a 50 mL centrifuge tube. Add 4 mL of a benzene:petroleum ether (1:1 *v*/*v*) mixed solvent, seal the tube, and let it stand for extraction for 24 h (keep the cap closed). Then add 4 mL of 0.4 mol/L potassium hydroxide:methanol solution, vortex for 3 min, and let stand for 30 min. Add 5 mL of ultrapure water to separate the layers (the lipid layer is on top, the aqueous layer in the middle, and the impurities at the bottom). Collect the upper layer, add an appropriate amount of anhydrous sodium sulfate (to absorb residual water). Take 500 μL of the prepared sample, dilute with 500 μL of hexane, filter through a 0.22 μm membrane, and inject. Chromatographic conditions: column, SP-2560 (100 m × 0.25 mm × 0.2 μm); carrier gas, high-purity nitrogen at a flow rate of 0.8 mL/min; injection volume, 1 μL with a split ratio of 20:1; FID detector temperature, 280 °C; hydrogen flow, 30 mL/min; air flow, 400 mL/min; and column temperature program: initial temperature 140 °C held for 5 min, then ramped at 3 °C/min to 220 °C and held for 40 min. The fatty acid composition of the LT was based on peak areas of the internal standards (methyl undecanoate, C11:0), and identified from mixed FAME standards (CRM47885, Sigma, Saint Louis, Darmstadt, Germany) [19].

### 2.6. Hydrolyzed Amino Acids

The content of hydrolyzed amino acids was determined using an automatic amino acid analyzer (LA8080, Hitachi, Tokyo, Japan) equipped with a post-column ninhydrin derivatization system. The freeze-dried samples were the same as in Section 2.5. Freeze-dried LT muscle samples (0.1 g) were weighed into hydrolysis tubes, and 15 mL of 6 mol/L HCl was added. The tubes were sealed using an alcohol blast burner (02075, Shandong Wantai Teaching Equipment Co., Ltd., Qingdao, China) and hydrolyzed at a constant temperature of 110 °C for 22 h. After cooling, the hydrolysis tubes were opened, and the hydrolysates were filtered and brought to a final volume of 50 mL. A 1 mL aliquot of the filtrate was evaporated to dryness, reconstituted in 1 mL of 0.02 mol/L HCl, passed through a 0.22 μm membrane filter, and collected as 200 μL in a sample vial for analysis [18].

### 2.7. Physiological and Biochemical Parameters

Serum concentrations of glucose [GLU, F00611 assay kit (glucose oxidase method)], as well as the activities of alanine aminotransferase [ALT, C01021 assay kit (trace method)], aspartate aminotransferase [AST, C00911 assay kit (trace method)], total superoxide dismutase [T-SOD, A00112 assay kit (Hydroxylamine method)], glutathione peroxidase [GSH-Px, A00521 assay kit (trace method)], and the levels of malondialdehyde [MDA, A00312 assay kit (TBA method)] and total antioxidant capacity [T-AOC, A01531 assay kit (FRAP method)] were determined using a microplate reader (Infinite M PLEX, Tecan, Salzburg, Austria). In addition, LT muscle samples (1 g) were homogenized in PBS (1 mL) using a homogenizer (JXFSTPRP-24, Shanghai Jingxin Industrial Development Co., Ltd., Shanghai, China). The homogenate was then centrifuged at 2000 rpm for 10 min at 4 °C. Subsequently, the supernatant was collected to assess the activities of lactate dehydrogenase [LDH, A02022 assay kit (trace method)], succinate dehydrogenase [SDH, A02211 assay kit (colorimetric method)], malate dehydrogenase [MDH, A02121 assay kit (colorimetric method)], T-SOD, GSH-Px, insulin-like growth factor-1 [IGF-1, H04112 assay kit (trace method)], and cytochrome coxidase [COX, H21011 assay kit (trace method)], as well as the levels of MDA and T-AOC, which were determined using a microplate reader (Infinite M PLEX, Tecan, Austria) and UV-Vis spectrophotometer (UV-3600Plus, Shimadzu, Kyoto, Japan). All assay kits were purchased from Nanjing Jiancheng Bioengineering Institute, Nanjing, China, and all procedures were performed strictly according to the manufacturer’s instructions.

### 2.8. Real-Time Quantitative Fluorescence

Total RNA was extracted from LT muscle samples using an RNA extraction kit (Steady Pure). Complementary DNA (cDNA) was synthesized using a reverse transcription kit (Evo M-MLV). Relative mRNA expression levels were detected via SYBR Green dye-based quantitative PCR (AG11701). All kits were provided by Accurate Biotechnology (Hunan) Co., Ltd., Changsha, China. A 10 µL reaction system was used, consisting of 5 µL of 2 × Premix, 0.2 µL each of forward and reverse primers, 1 µL of cDNA template, and sterile water to a final volume of 10 µL. The thermal cycling protocol was carried out strictly according to the manufacturer’s instructions. Gene expression levels were normalized to the reference gene *GAPDH* and analyzed using the 2^−ΔΔCt^ method; normalization was performed according to Ma et al. [20]. Primers were synthesized in Beijing Qingke Biotechnology Co., Ltd., Beijing, China and their sequences can be found in Table S2.

### 2.9. Statistical Analysis

Data were organized in Excel, and all statistical analyses were performed using SPSS 26. The Shapiro–Wilk test and Levene’s test were used to assess normality and homogeneity of variances, respectively. Outliers were examined by the Grubbs test (α = 0.05); when an outlier was identified with compelling biological or technical justification, it was removed. Carcass traits and meat quality indicators were compared among the three groups using one-way analysis of variance (ANOVA). When ANOVA yielded a significant result, followed by Duncan’s multiple range test to evaluate between-group differences. Each individual pig served as the experimental unit (*n* = 6 per group). Results are expressed as mean ± standard deviation (SD). For subsequent investigations, the most appropriate dose group was identified based on the above outcomes; no additional *t*-test was applied to the same set of carcass and meat quality data that had already undergone multi-group comparison. All figures were generated with GraphPad Prism 10, and differences were considered statistically significant at *p* < 0.05.

## 3. Results

### 3.1. FA Improved Carcass Traits

As shown in Table 1, the FA reduced the fat percentage and backfat thickness, and increased the lean meat percentage and loin-eye area of Shaziling pigs (*p* < 0.05), but had no significant effect on carcass weight or dressing carcass yield (*p* > 0.05). Compared with the control group, the 250 mg/kg FA group exhibited a decrease in fat percentage (*p* < 0.05). Compared with the control group, supplementation with 250 mg/kg FA increased the lean meat percentage and loin-eye area of Shaziling pigs, whereas supplementation with 500 mg/kg FA had no significant effect on loin-eye area (*p* > 0.05), but increased the lean meat percentage (*p* < 0.05). All in all, the carcass traits of Shaziling pigs in the 250 mg/kg FA group was superior to that of the control group and the 500 mg/kg FA group.

### 3.2. FA Improved Meat Quality

As shown in Table 2, the FA increased the redness of the LT muscle at 45 min and 24 h postmortem, and improved the pH at 45 min postmortem and crude protein content of Shaziling pigs (*p* < 0.05), but had no significant effect on shear force, drip loss, lightness, yellowness, moisture content, crude fat content, and cooking percentage of the LT muscle (*p* > 0.05). The 250 mg/kg FA group showed higher redness values at 45 min and 24 h postmortem and a higher pH at 45 min postmortem in the LT muscle compared with the control group and the 500 mg/kg FA group (*p* < 0.05). However, no significant differences were observed in meat color or pH values of the LT muscle between the control group and the 500 mg/kg FA group (*p* > 0.05). The crude protein content of the LT muscle in the 250 mg/kg FA group was higher than that in the control group and the 500 mg/kg FA group (*p* < 0.05). In summary, the meat quality of Shaziling pigs in the 250 mg/kg FA group was superior to that of the control group and the 500 mg/kg FA group.

### 3.3. FA Increased the Amino Acid Content

Based on the experimental results presented in Section 3.1 and Section 3.2, the control group and the 250 mg/kg FA group of Shaziling pigs were selected for further investigation. As shown in Table 3, dietary supplementation with 250 mg/kg FA increased the methionine content in the LT muscle of Shaziling pigs (*p* < 0.05), but had no significant effect on the content or composition of the other hydrolyzed amino acids (*p* > 0.05).

### 3.4. FA Modulated Fatty Acid Content

As shown in Table 4, dietary supplementation with 250 mg/kg FA increased the capric acid content and decreased the stearic acid content in the LT muscle of Shaziling pigs (*p* < 0.05), but had no significant effect on the content or composition of the other medium- and long-chain fatty acids (*p* > 0.05).

### 3.5. FA Enhances the Antioxidant Capacity

As shown in Table 5, 250 mg/kg FA reduced the serum GLU concentration in Shaziling pigs (*p* < 0.05), but had no significant effect on serum AST or ALT (*p* > 0.05). In the LT muscle, 250 mg/kg FA increased MDH activity, and IGF-1 content, while decreasing LDH activity (*p* < 0.05). However, it had no significant effect on SDH or COX activity (*p* > 0.05). Adding 250 mg/kg FA increased T-SOD activity in both the LT muscle and serum, and decreased MDA content (*p* < 0.05). Additionally, the FA increased serum GSH-Px peroxidase activity (*p* < 0.05), but had no significant effect on T-AOC (*p* > 0.05).

### 3.6. FA Altered the Morphology of the LT Muscle

**Figure 1:**
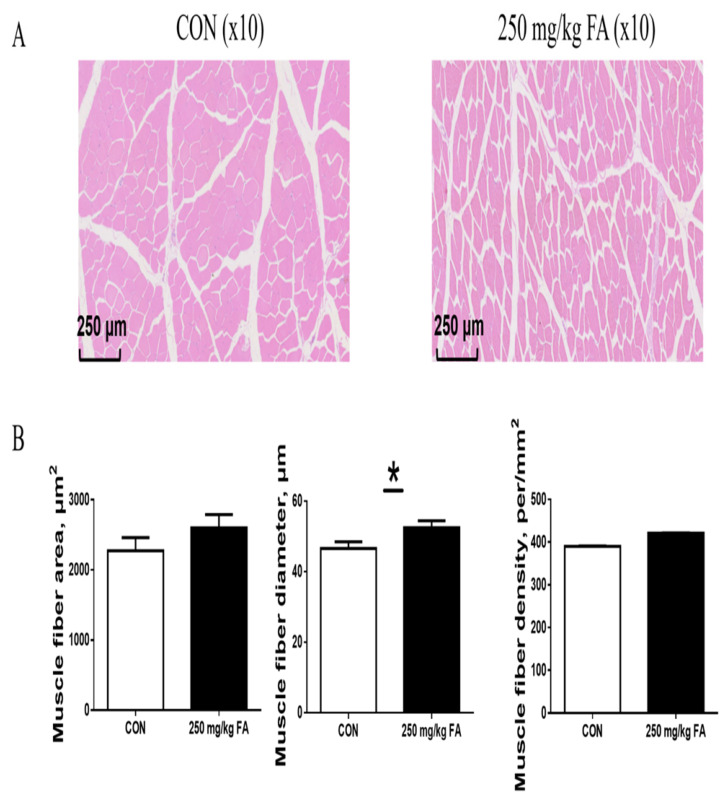
Effect of FA on the morphology of the LT muscle in Shaziling pigs. (**A**) H&E staining of the LT muscle. (**B**) Visualization of LT muscle fiber diameter, cross-sectional area, and density. * indicates *p* < 0.05, which was considered statistically significant. CON = basal diet; 250 mg/kg FA = basal diet + 250 mg/kg ferulic acid.

The H&E staining analysis revealed no inflammation in the LT muscle of Shaziling pigs in either the control group or the FA group, and the tissue morphology remained intact (Figure 1A). Further analysis showed that FA increased the muscle fiber diameter in the LT of Shaziling pigs (*p* < 0.05; Figure 1B), but had no significant effect on muscle fiber density or muscle fiber area (*p* > 0.05; Figure 1B).

### 3.7. FA Alters Gene Expression Related to Muscle Fiber Type and Development

**Figure 2:**
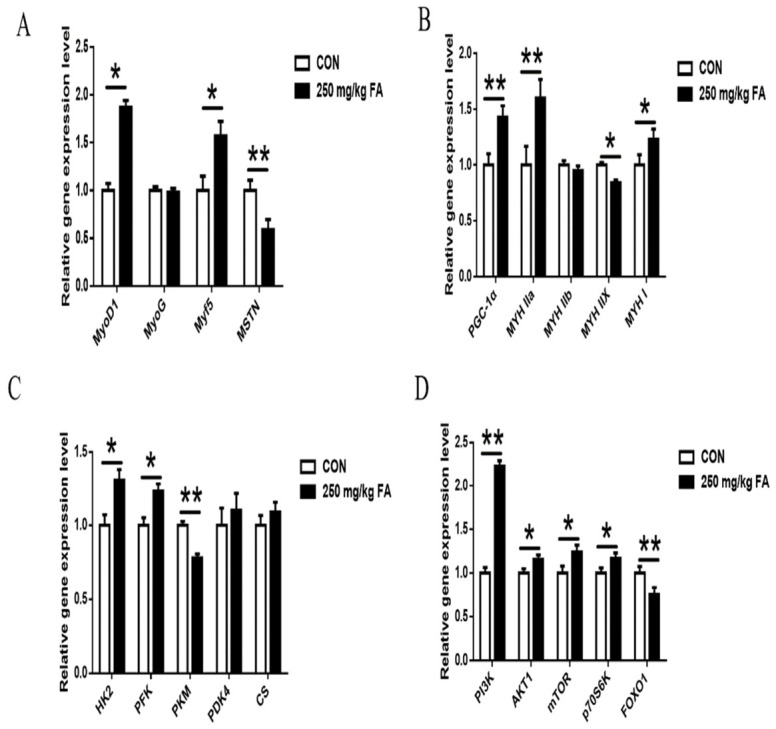
Effect of FA on the expression of genes related to meat quality and muscle fiber development in Shaziling pigs. (**A**): Muscle development-related genes; (**B**): Muscle fiber type-related genes, (**C**): Glucose metabolism-related genes; (**D**): Protein synthesis and degradation (PI3K/AKT1/mTOR) signaling pathway-related genes; * indicates *p* < 0.05 and ** indicates *p* < 0.01, which were considered statistically significant. CON = basal diet; 250 mg/kg FA = basal diet + 250 mg/kg ferulic acid.

As shown in Figure 2, analysis of genes related to muscle development revealed that 250 mg/kg FA increased the expression of *PI3K*, *AKT1*, *mTOR*, *p70S6K*, *MyoD1*, and *Myf5* genes, while decreasing the expression of *FOXO1* and *MSTN* genes in the LT muscle of Shaziling pigs (*p* < 0.05). However, it had no significant effect on the expression of the *MyoG* gene (*p* > 0.05). Analysis of genes related to meat quality and glycometabolism showed that 250 mg/kg FA increased the expression of *HK2*, *PFK*, *PGC-1α*, *MYH IIa*, and *MYH I* genes, while decreasing the expression of *PKM* and *MYH IIX* genes in the LT muscle of Shaziling pigs (*p* < 0.05). However, it had no significant effect on the expression of *MYH IIb*, *CS* and *PDK4* genes (*p* > 0.05).

## 4. Discussion

Chinese indigenous pig breeds, such as the Shaziling pig, are renowned for their excellent meat quality and distinctive flavor. However, they generally exhibit a low lean meat percentage and excessive backfat thickness, which limit their market competitiveness [4]. Improving carcass composition without compromising superior meat quality is a critical issue that must be addressed in the development and utilization of FA for meat quality and muscle development in the Shaziling pig. Our findings demonstrate that FA exerts positive regulatory effects on carcass traits, meat quality, and skeletal muscle physiology primarily by modulating antioxidant defense, promoting myofiber type switching, and modulating key anabolic signaling pathways. Specifically, dietary supplementation with 250 mg/kg FA significantly reduced fat percentage and backfat thickness while increasing lean meat percentage and loin-eye area in Shaziling pigs. This nutrient repartitioning effect, which favors lean tissue deposition and suppresses fat accumulation, is consistent with the findings of Valenzuela-Grijalva et al. [4] in commercial finishing pigs, where FA supplementation increased average daily gain and loin-eye area while reducing fat deposition. In the present study, the 500 mg/kg FA treatment did not further improve these traits, suggesting potential pro-oxidant effects at high FA concentrations [21]. Additionally, our phenotypic data analysis showed that supplementing the basal diet of Shaziling pigs with 250 mg/kg FA was overall superior to the 500 mg/kg dose, as evidenced by more favorable changes in fat percentage, lean meat percentage, 45 min pH of the LT muscle, and 24 h redness value. Therefore, the 250 mg/kg FA group and the control group were selected for further investigation. However, whether this effect is dose-dependent requires further study. Notably, Because the 250 mg/kg FA group showed superior carcass and meat quality traits, subsequent analyses were performed only for the CON and 250 mg/kg FA groups. This post hoc selection is acknowledged as a limitation, and the results should be interpreted as exploratory.

In addition, the FA increased muscle redness and pH at 45 min postmortem, consistent with findings from other studies showing that FA partially improves these indices through enhanced postmortem glycolytic regulation and myoglobin stability [22,23]. Significant elevations in T-SOD and GSH-Px activities in serum and muscle, coupled with a concurrent decrease in MDA content, provide robust evidence that FA exerts potent antioxidant functions in vivo. As a natural phenolic compound, FA can directly scavenge free radicals and likely upregulates endogenous antioxidant defense systems [23]. The alleviation of oxidative stress is crucial for maintaining postmortem muscle integrity and color stability. Concurrently, increased MDH activity and decreased LDH activity indicate a metabolic reprogramming from glycolysis toward oxidative metabolism in muscle, which helps prevent an excessively rapid postmortem pH decline and improves water-holding capacity [24]. Notably, the present study found that *HK2* and *PFK* expression were upregulated, whereas *PKM* expression was downregulated, indicating that FA enhances the initial steps of glucose uptake and phosphorylation in muscle to meet the increased oxidative metabolic demand of converted myofibers, while simultaneously suppressing the terminal step that leads to lactate accumulation [4,10,23]. This coordinated gene expression pattern optimizes postmortem muscle energy metabolism, contributing to a stable pH decline and improved meat quality.

Histological analysis confirmed that FA increased myofiber diameter without inducing inflammation. This finding contrasts with the results of Valenzuela-Grijalva et al. [4], who observed reduced myofiber cross-sectional area and increased fiber number per unit area. These discrepancies may stem from differences in pig breed, FA dosage, and feeding duration [25]. Critically, we found that FA induced a favorable shift in myofiber type toward oxidative fibers by upregulating *MYH I* and *MYH IIa* while downregulating *MYH IIx* expression. This shift is likely mediated by the upregulation of *PGC-1α*, the master regulator of oxidative fiber formation. This result aligns with the findings of Wang et al. [10], who first demonstrated that FA promotes the transition from fast-twitch to slow-twitch myofibers in skeletal muscle of weaned piglets via the SIRT1/AMPK/PGC-1α signaling pathway. This transition toward an oxidative muscle phenotype is directly associated with the improved meat color and pH observed in this study.

Meanwhile, our findings showed that elevated IGF-1 content further confirms an enhanced anabolic state in muscle, likely a downstream effect of improved cellular health and signaling [26]. Notably, a key finding of this study is the modulation of the PI3K/AKT/mTOR signaling axis by FA. Upregulation of *PI3K*, *AKT1*, *mTOR*, and *p70S6K*, as well as the myogenic regulatory factors *MyoD1* and *MyoG*, along with downregulation of the negative regulators *FOXO1* and *MSTN*, provides a clear molecular mechanism for the observed muscle hypertrophic effect. This pathway serves as a central hub that integrates nutritional and endocrine signals to promote skeletal muscle protein synthesis while inhibiting protein degradation [4,27]. The downregulation of *MSTN* expression is particularly noteworthy, as it represents a potential mechanism for enhancing lean growth without exogenous hormonal intervention [28,29]. Although direct evidence in pigs is still limited, the present findings suggest the notion that FA acts as a natural anabolic agent maybe by targeting this key signaling cascade. Furthermore, this study identified significant increases in methionine content and alterations in capric acid and stearic acid levels, these findings suggest that FA may influence specific amino acid and fatty acid profiles. Methionine is one of the limiting amino acids in pigs and is essential for protein synthesis and as a methyl donor [30]. Its increased content may be attributed to enhanced protein synthesis efficiency and improved oxidative protection [27,30]. The changes in fatty acid composition suggest that FA might affect the activities of enzymes such as fatty acid synthase and stearoyl-CoA desaturase, thereby potentially enhancing the eating quality and nutritional value of pork; however, direct measurements of these enzyme activities are needed to confirm this speculation [31,32].

## 5. Conclusions

In conclusion, our study demonstrates that dietary supplementation with 250 mg/kg FA effectively improves meat quality and muscle development in Shaziling pigs. FA acts as a potent antioxidant, increases muscle fiber diameter, and promotes a shift toward oxidative muscle fibers. Importantly, it also upregulates genes of the PI3K/AKT1/mTOR signaling cascade (*PI3K*, *AKT1*, *mTOR*, *p70S6K*), promoting protein synthesis and muscle development while inhibiting negative regulators (*FOXO1*, *MSTN*). Furthermore, FA favorably modulates amino acid and fatty acid profiles and optimizes postmortem glucose metabolism. These findings provide a solid theoretical basis for the application of FA as a natural, functional feed additive to enhance both production efficiency and pork quality in Shaziling and other local pig populations. Future studies should include protein-level validation of the key signaling pathways (e.g., Western blotting for PI3K, AKT, mTOR) and further investigate the dose-dependent effects of FA to determine the optimal supplementation level for indigenous pig breeds.

## Figures and Tables

**Table 1:** Effects of FA on carcass traits in Shaziling pigs.

Items	CON	250 mg/kg FA	500 mg/kg FA	*p*-Value
Carcass weight, kg	54.04 ± 4.77	57.67 ± 5.24	54.51 ± 4.59	0.324
Carcass yield, %	72.41 ± 1.78	72.83 ± 1.39	72.18 ± 1.17	0.962
Fat percentage, %	36.02 ± 2.38 ^a^	33.61 ± 0.83 ^b^	34.23 ± 0.86 ^ab^	0.032
Lean meat percentage, %	42.05 ± 1.28 ^c^	44.83 ± 0.86 ^a^	43.49 ± 0.35 ^b^	<0.001
Loin-eye area, cm^2^	18.25 ± 1.98 ^b^	22.31 ± 2.43 ^a^	20.65 ± 2.66 ^ab^	0.018
Backfat thickness, cm	34.44 ± 2.01 ^a^	28.37 ± 0.67 ^b^	33.77 ± 1.10 ^a^	0.001

Different lowercase superscript letters indicate significant differences. CON = basal diet; 250 mg/kg FA = basal diet supplemented with 250 mg/kg ferulic acid; 500 mg/kg FA = basal diet supplemented with 500 mg/kg ferulic acid.

**Table 2:** Effect of FA on the meat quality in Shaziling pigs.

Items	CON	250 mg/kg FA	500 mg/kg FA	*p*-Value
Shear force, N	10.54 ± 0.71	9.70 ± 2.72	10.74 ± 0.75	0.806
Drip loss, %	0.73 ± 0.10	0.77 ± 0.27	0.77 ± 0.26	0.329
Water loss ratio, %	13.26 ± 1.60	13.34 ± 1.00	14.00 ± 1.48	0.606
Cooking percentage, %	64.12 ± 1.72	65.39 ± 2.35	66.08 ± 2.39	0.229
pH _45min_	6.13 ± 0.18 ^b^	6.47 ± 0.27 ^a^	6.41 ± 0.14 ^ab^	0.026
pH _24h_	5.99 ± 0.14	6.08 ± 0.24	6.09 ± 0.03	0.826
Brightness, L*_45min_	40.26 ± 2.38	40.05 ± 1.44	40.53 ± 1.87	0.911
Redness, a*_45min_	6.50 ± 0.88 ^b^	8.26 ± 1.88 ^a^	7.05 ± 0.30 ^b^	0.010
Yellowness, b*_45min_	2.96 ± 1.21	2.31 ± 0.73	3.01 ± 0.60	0.335
Brightness, L*_24h_	41.07 ± 2.72	44.05 ± 3.44	42.73 ± 3.52	0.311
Redness, a*_24h_	7.43 ± 1.19 ^b^	11.03 ± 2.88 ^a^	9.41 ± 0.59 ^ab^	0.013
Yellowness, b*_24h_	6.05 ± 0.06	6.56 ± 0.97	6.31 ± 0.81	0.372
Moisture, %	72.10 ± 0.76	72.67 ± 0.48	73.00 ± 0.71	0.089
Crude protein, %	24.44 ± 0.72 ^b^	25.68 ± 0.71 ^a^	24.59 ± 0.51 ^b^	0.007
Crude fat, %	2.59 ± 0.43	2.32 ± 0.41	2.43 ± 0.24	0.463

Different lowercase superscript letters indicate significant differences. CON = basal diet; 250 mg/kg FA = basal diet + 250 mg/kg ferulic acid; 500 mg/kg FA = basal diet + 500 mg/kg ferulic acid.

**Table 3:** Effects of FA on the hydrolyzed amino acids of the LT in Shaziling pigs (g/100 g).

Items	CON	250 mg/kg FA	*p*-Value
Asp	4.42 ± 0.20	4.39 ± 0.07	0.738
Thr	2.19 ± 0.10	2.18 ± 0.03	0.940
Ser	1.86 ± 0.09	1.85 ± 0.02	0.832
Glu	6.94 ± 0.30	6.93 ± 0.08	0.901
Gly	2.07 ± 0.12	2.00 ± 0.03	0.184
Ala	2.66 ± 0.12	2.63 ± 0.04	0.620
Cys	0.41 ± 0.02	0.40 ± 0.01	0.164
Val	2.39 ± 0.10	2.37 ± 0.04	0.590
Met	0.86 ± 0.05	1.19 ± 0.15	0.002
Ile	2.30 ± 0.10	2.29 ± 0.03	0.712
Leu	3.91 ± 0.18	3.89 ± 0.05	0.800
Tyr	1.66 ± 0.07	1.69 ± 0.04	0.339
Phe	2.53 ± 0.12	2.51 ± 0.06	0.748
Lys	4.20 ± 0.19	4.19 ± 0.06	0.874
His	2.36 ± 0.13	2.34 ± 0.09	0.765
Arg	3.07 ± 0.13	3.05 ± 0.04	0.779
Pro	1.78 ± 0.09	1.76 ± 0.02	0.579
TAA	45.60 ± 2.03	45.63 ± 0.61	0.978
EAA	21.52 ± 0.91	21.59 ± 0.32	0.868
NEAA	21.75 ± 1.01	21.48 ± 0.29	0.561
BCAA	8.61 ± 0.38	8.54 ± 0.12	0.688
SAA	8.78 ± 0.41	8.66 ± 0.11	0.526
FLAA	11.36 ± 0.50	11.31 ± 0.14	0.829

CON = basal diet; 250 mg/kg FA = basal diet + 250 mg/kg ferulic acid; TAA = total amino acids; EAA = essential amino acids; BCAA = branched chain amino acids; NEAA = non-essential amino acids; SAA = sweet tasting; FLAA = flavor-enhancing amino acids.

**Table 4:** Effect of FA on medium- and long-chain fatty acid composition in the LT in Shaziling pigs (%).

Items	CON	250 mg/kg FA	*p*-Value
C6:0	0.12 ± 0.06	0.14 ± 0.05	0.586
C8:0	0.40 ± 0.17	0.47 ± 0.15	0.500
C10:0	0.19 ± 0.02	0.25 ± 0.05	0.030
C12:0	0.07 ± 0.01	0.08 ± 0.01	0.560
C14:0	1.29 ± 0.21	1.24 ± 0.19	0.683
C15:0	0.22 ± 0.09	0.20 ± 0.02	0.524
C16:0	25.19 ± 1.21	25.09 ± 1.19	0.887
C16:1	3.12 ± 0.50	3.56 ± 0.70	0.243
C17:0	0.16 ± 0.01	0.15 ± 0.01	0.063
C17:1	0.10 ± 0.01	0.10 ± 0.01	0.116
C18:0	13.15 ± 0.42	12.20 ± 0.32	0.001
C18:1n9c	39.64 ± 2.53	38.77 ± 1.19	0.496
C18:1n9t	0.16 ± 0.01	0.18 ± 0.03	0.401
C18:2n6c	11.71 ± 2.45	12.49 ± 2.34	0.584
C18:2n6t	0.09 ± 0.05	0.05 ± 0.01	0.112
C18:3n6	0.06 ± 0.02	0.07 ± 0.02	0.230
C18:3n3	0.38 ± 0.05	0.35 ± 0.03	0.154
C20:0	0.24 ± 0.03	0.23 ± 0.02	0.277
C20:1	0.75 ± 0.12	0.64 ± 0.06	0.078
C20:3n6	0.31 ± 0.13	0.37 ± 0.10	0.409
C20:4n6	2.16 ± 0.09	2.64 ± 0.78	0.351
C20:2	0.39 ± 0.08	0.44 ± 0.09	0.362
C21:0	0.05 ± 0.01	0.05 ± 0.01	1.000
C22:0	0.10 ± 0.03	0.10 ± 0.03	0.783
C23:0	0.08 ± 0.03	0.09 ± 0.03	0.543
SFA	41.52 ± 1.22	39.43 ± 1.09	0.199
MUFA	43.77 ± 2.79	43.24 ± 2.06	0.717
PUFA	15.10 ± 3.51	16.40 ± 3.13	0.512

CON = basal diet; 250 mg/kg FA = basal diet + 250 mg/kg ferulic acid; MUFA = monounsaturated fatty acid; SFA = saturated fatty acid; PUFA = polyunsaturated fatty acid.

**Table 5:** Effects of FA on physiological and biochemical profile in Shaziling pigs.

Items	CON	250 mg/kg FA	*p*-Value
Serum			
GLU, mmol/L	5.94 ± 0.64	5.09 ± 0.61	0.028
AST, U/L	0.10 ± 0.01	0.10 ± 0.01	0.143
ALT, U/L	0.02 ± 0.00	0.02 ± 0.00	0.946
GSH-Px, U/mL	8627.87 ± 583.56	9427.03 ± 214.14	0.010
T-SOD, U/mL	63.72 ± 3.52	71.72 ± 4.76	0.008
T-AOC, mmol/L	0.30 ± 0.05	0.30 ± 0.02	0.833
MDA, nmol/mL	0.10 ± 0.03	0.06 ± 0.02	0.014
LT			
LDH, U/gprot	8.56 ± 0.14	7.52 ± 0.05	<0.001
SDH, U/mgprot	2.66 ± 0.35	3.02 ± 0.51	0.188
MDH, U/mgprot	0.17 ± 0.02	0.20 ± 0.01	0.044
COX, U/gprot	212.74 ± 10.79	215.54 ± 10.73	0.662
IGF-1, ng/gprot	116.01 ± 4.29	133.74 ± 13.10	0.010
GSH-Px, U/mgprot	40.41 ± 3.27	44.61 ± 6.42	0.184
T-SOD, U/mgprot	147.38 ± 3.61	165.93 ± 10.04	0.002
T-AOC, mmol/gprot	0.02 ± 0.00	0.02 ± 0.00	0.879
MDA, nmol/mgprot	5.37 ± 0.30	4.51 ± 0.34	0.001

CON = basal diet; 250 mg/kg FA = basal diet + 250 mg/kg ferulic acid; ALT = alanine aminotransferase; GSH-Px = glutathione peroxidase; AST = aspartate aminotransferase; MDA = malondialdehyde; T-AOC = total antioxidant capacity; LDH = lactate dehydrogenase; GLU = glucose; SDH = succinate dehydrogenase; IGF-1 = insulin-like growth factor 1; MDH = malate dehydrogenase; COX = cytochrome c oxidase; T-SOD = total superoxide dismutase.

## Data Availability

The original contributions presented in the study are included in the article/Supplementary Materials; further inquiries can be directed to the corresponding authors.

## Supplementary Materials

The following supporting information can be downloaded at: https://www.mdpi.com/article/10.3390/foods15122111/s1, Table S1: Composition and nutrient levels of basal diets (air-dry basis); Table S2: Primer sequence.
