# Supplementary material for: Effects of Ferulic Acid on Meat Quality, Carcass Traits, Muscle Fiber Types, and Muscle Development in Shaziling Pigs
Source: Foods. 2026 Jun 11;15(12):2111. doi: 10.3390/foods15122111 (PMC13298282; doi:10.3390/foods15122111)
Supplement: Supplementary file 1 [file foods-15-02111-s001.zip › foods-4354586-supplementary.pdf]

Table S1

Composition and nutrient levels of basal diets (air-dry basis) %

Ingredient	30-50 kg	50-80 kg
Corn (> 8%)	69.77	69.77
Wheat bran	11.12	13.14
Soybean oil	0.81	0.61
Soybean meal (> 43%)	14.16	10.11
Bran flour	1.21	3.44
NaCl	0.36	0.40
Calcium hydrogen phosphate	0.61	0.46
Choline chloride (50%)	0.09	0.08
Mountain flour	1.32	1.42
L-Lysine HCl (98%)	0.22	0.24
L-methionine (98%)	0.10	0.11
L-Threonine (98%)	0.08	0.10
Swine vitamin premix [1]	0.12	0.10
Swine mineral premix [2]	0.03	0.02
Nutrient levels [3]		
Digestible energy, MJ/kg	13.18	12.73
Crude protein	13.48	12.03
Total calcium	0.71	0.69
Total phosphorus	0.58	0.53
Effective phosphorus	0.27	0.24
Lysine	0.77	0.69
Methionine	0.31	0.30
Methionine+cysteine	0.54	0.51
Threonine	0.57	0.53
Tryptophan	0.14	0.12

[1] Minimum provided per kg of diet: 11000 IU Vitamin A, 1650 IU Vitamin D_3, 55 IU Vitamin E, 0.044 mg Vitamin B_{12}, 4.4 mg Menadione, 0.17 mg Biotin, 1.1 mg Folic Acid, 60.5 mg Niacin, 0.05 mg d-Pantothenic Acid, 3.3 mg Vitamin B6, 9.9 mg Riboflavin, 3.3 mg Thiamine.

[2] Minimum provided per kg of diet: 150 mg Fe, 165 mg Zn, 44.1 mg Mn, 16.5 mg Cu, 0.5 mg I, 0.3 mg Co, and 0.03 mg Se.

[3] The nutritional level of feed is calculated.

Table S2

Primer sequence

Genes	Sequence（5′→3′）	Accession number
PI3K	F:GCTGTGCTGGATATTGCGTG<br>R:GGACTGCCTCTTCATCGGAC	NM_001012956.2
AKT1	F:TCGCCCCTCAACAACTTCTC<br>R:AGGGACACCTCCATCTCCTC	XM_021081501.1
mTOR	F:TGATTTGGTTCCCAGGACGG<br>R:CTGAGTCGCTGTGTCCTTGT	XM_003127584.6
p70S6K	F:AAACACCTGTTGACAGCCCA<br>R:GTGCTCTGGTCGTTTGGAGA	XM_021067294.1
FOXO1	F:AGGATAAGGGCGACAGCAAC<br>R:GGGAGAGAGCCTCCCACTAA	NM_214014.3
MSTN	F:CGCCTGGAAACAGCTCCTAA<br>R:AGGAGTCTTGACGGGTCTCA	NM_214435.2
MyoD1	F:CCTAAAGCCCGAGGAACACT<br>R:TCGAAGGCCTCGTTGACTTT	NM_001002824.1
MyoG	F:GAAAACTACCTGCCCGTCCA<br>R:CCACAGACACGGACTTCCTC	NM_001012406.1
Myf5	F:GACGAGTTTGAGCCACGAGT<br>R:GTGGATTTCCTCTTGCACGC	NM_001278775.1
*HK*2	F:CAGGAGATCGACATGGGCTC<br>R:GTTCAGGACTGAGCTTCCCC	NM_001122987.1
PFK	F:GGAGAGCTGAGACTATAAGAGTGG<br>R:GTTATCTCCGCCATCCACCA	NM_001044550.1
PKM	F:GCCAAACCAAGCAGCAACA<br>R:GCTCTGTTGTGGTGGTGTGTGTGTA	XM_021099117.1
PGC-1α	F:ATGTGCAACCAGGACTCTGTA<br>R:GGCAATCCGTCTTCATCCAC	NM_213963.2
MYH I	F:CAGACGGAAGAGGACAGGAAG<br>R:AATCTACTCCTCGTTCAAGCCC	NM_213855.2
MYH IIa	F:GTTCAAGCAGAAGCAACGGG<br>R:ACGGATTTGGAGAGTGCGTT	XM_013981330.2
MYH IIb	F:CCAGGGAGAGATGGAGGACA<br>R:TCAAGTTCACGTACCCTGGC	NM_001104951.2
MYH IIX	F:GCTGAGATGCCTCTGTCCTG<br>R:TTTCGGAGGTAAGGAGCAGC	XM_021066035.1
CS	F:GGCTCTTCGGAGCCAAGAA<br>R:CTTCCCCACCCTTAGCCTTG	NM_214276.1
PDK4	F:TCCTACGACTCAGTGCCTCA<br>R:GCTTTATGGACAGCGGGGAT	NM_001159306.1
GAPDH	F:CTGCTCCTCCCCGTTCGAC<br>R:GACAGCCGTGTGTTCCGT	XM_021091114.1
